# Supplementary material for: Molecular Genetics and Functional Analysis Implicate CDKN2BAS1-CDKN2B Involvement in POAG Pathogenesis
Source: Cells. 2020 Aug 21;9(9):1934. doi: 10.3390/cells9091934 (PMC7564117; doi:10.3390/cells9091934)
Supplement: Supplementary file 1 [file cells-09-01934-s001.pdf]

Supplementary Materials:

Supplementary Table S1: Primers sequence used for real-time RT-PCR analysis of *CDKN2B-AS1*, *CDKN2B*, *CDKN2A* and *p14ARF* genes in human retina and HEK293T and TM cells. F=Forward primer, R=Reverse primer.

Gene	Sequence 5' to 3'
CDKN2B-AS1	F: TGCCTGCCCTGTCGAGGAACA
	R: AAGCAGTACTGACTCGGGAAAGGA
CDKN2B	F: TTTCGGGAGGCGCGCGATC
	R: GGTGCTCTGCAGCGTCGTGA
CDKN2A	F: TTACGGTCGGAGGCCGATCCA
	R: GAGGGACCTTCCGCGGCATC
p14ARF	F: AGCAGCCGCTTCCTAGAAGACCA
	R: AGGGACCTTCCGCGGCATCT

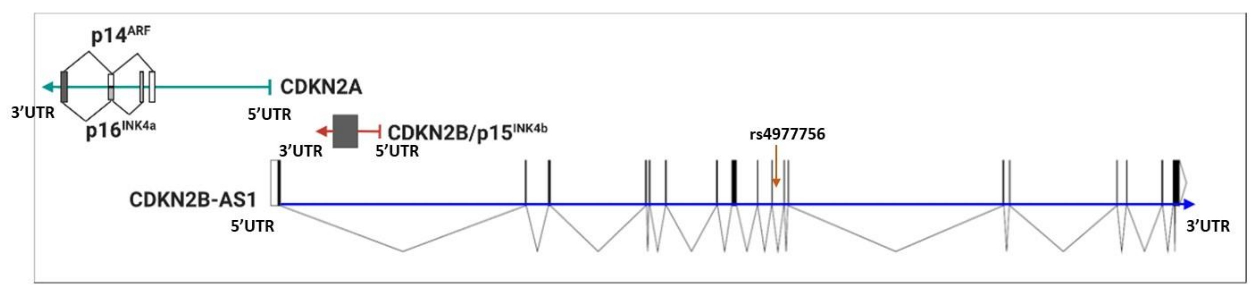

Supplementary Figure 1: Schematic representation showing *CDKN2B-AS1*, *CDKN2B* and *CDKN2A* gene and location of rs4977756 (indicated by arrowhead) within *CDKN2B-AS1* gene.

Supplementary Table S2: Comprehensive list SNPs associated with POAG in 9p21 locus in diverse populations.

SNP ID	Gene	Population (Cases/ controls)	Association with Phenotype/Traits	Associated allele	MAF in cases/controls	References
rs1063192	*CDKN2B*	African Caribbean (272/165)	POAG	C	0.039/0.094	[1]

		American (539/336)	POAG, VCDR	G	0.342/0.417	[2]
		Chinese (1157/934)	POAG	C	0.184/0.204	[3]
		Australian (326/883)	Advanced OAG, VCDR	G	0.33/0.44	[4]
		Japanese (425/191)	NTG, VCDR	T	0.138/0.223	[5]
rs518394	*CDKN2B-AS1*	Japanese (740/2723)	POAG, RNFL	C	0.104/0.141	[6]
rs4977756	*CDKN2B-AS1*	Australian (892/4582)	OAG, advanced OAG	A	0.67/0.60	[7]
		Chinese (1157/934)	not associated with POAG	G	0.214/0.227	[3]
		Japanese (740/2723)	POAG, VFD, RNFLT	G	0.213/0.258	[6]
		African Caribbean 272/165	no association with POAG	G	0.33/0.358	[1]
rs2157719	*CDKN2B-AS1*	Chinese (1157/934)	POAG (HTG& NTG),IOP	G	0.092/0.133	[3]
		Saudi Arabian (85/95)	CDR	G	0.182/0.818	[8]
		Japanese (565/1104)	IOP	T	0.898/0.838	[9]
rs10120688	*CDKN2B-AS1*	Japanese (740/2723)	POAG	G	0.306/0.349	[6]
		Australian and New Zealand (334/434)	NTG, advanced POAG,VCDR, IOP	A	0.596/0.4856 (NTG),0.5372/ 0.4856	[10]
rs7049105	*CDKN2B-AS1*	Chinese (1157/934)	POAG (HTG& NTG)	A	0.319/0.361	[3]
		Australian and New Zealand (892/4582)	POAG (HTG& NTG), IOP, VCDR	G	0.5787/0.4609 (NTG), 0.5299/0.4609 (HTG)	[10]
rs523096	*CDKN2B-AS1*	Japanese (620/578)	NTG	A	0.825/0.904	[11]
		Chinese (1157/934)	POAG (HTG& NTG), IOP	C	0.095/0.135	[3]
Abbreviations: NTG- Normal Tension Glaucoma; HTG- High Tension Glaucoma; IOP- Intra Ocular Pressure; POAG- Primary Open-Angle Glaucoma; VCDR- Vertical Cup-Disc Ratio; CDR- Cup to Disc Ratio; RNFLT-Retinal Nerve Layer Thickness						

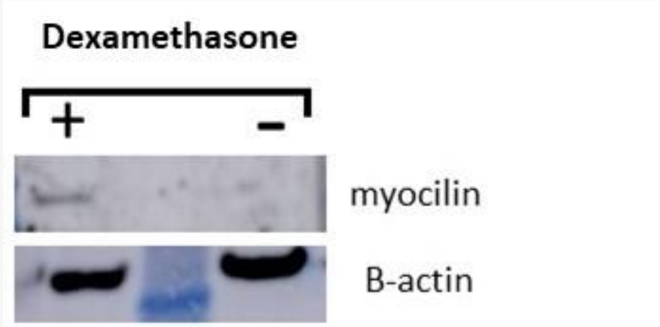

Supplementary Figure 2: Western blot showing the induction of Myocilin in TM cultures (P2 to P4 cultures) with 100mM Dexamethasone treatment for 3 days.

References:

1. Cao, D.; Jiao, X.; Liu, X.; Hennis, A.; Leske, M.C.; Nemesure, B.; Hejtmancik, J.F. CDKN2B polymorphism is associated with primary open-angle glaucoma (POAG) in the Afro-Caribbean population of Barbados, West Indies. *PLoS One* **2012**, *7*, e39278, doi:10.1371/journal.pone.0039278.
2. Fan, B.J.; Wang, D.Y.; Pasquale, L.R.; Haines, J.L.; Wiggs, J.L. Genetic variants associated with optic nerve vertical cup-to-disc ratio are risk factors for primary open angle glaucoma in a US Caucasian population. *Invest Ophthalmol Vis Sci* **2011**, *52*, 1788-1792, doi:10.1167/iovs.10-6339.
3. Chen, Y.; Hughes, G.; Chen, X.; Qian, S.; Cao, W.; Wang, L.; Wang, M.; Sun, X. Genetic Variants Associated With Different Risks for High Tension Glaucoma and Normal Tension Glaucoma in a Chinese Population. *Invest Ophthalmol Vis Sci* **2015**, *56*, 2595-2600, doi:10.1167/iovs.14-16269.
4. Dimasi, D.P.; Burdon, K.P.; Hewitt, A.W.; Fitzgerald, J.; Wang, J.J.; Healey, P.R.; Mitchell, P.; Mackey, D.A.; Craig, J.E. Genetic investigation into the endophenotypic status of central corneal thickness and optic disc parameters in relation to open-angle glaucoma. *Am J Ophthalmol* **2012**, *154*, 833-842 e832, doi:10.1016/j.ajo.2012.04.023.
5. Mabuchi, F.; Sakurada, Y.; Kashiwagi, K.; Yamagata, Z.; Iijima, H.; Tsukahara, S. Association between genetic variants associated with vertical cup-to-disc ratio and phenotypic features of primary open-angle glaucoma. *Ophthalmology* **2012**, *119*, 1819-1825, doi:10.1016/j.ophtha.2012.02.044.
6. Yoshikawa, M.; Nakanishi, H.; Yamashiro, K.; Miyake, M.; Akagi, T.; Gotoh, N.; Ikeda, H.O.; Suda, K.; Yamada, H.; Hasegawa, T., et al. Association of Glaucoma-Susceptible Genes to Regional Circumpapillary Retinal Nerve Fiber Layer Thickness and Visual Field Defects. *Invest Ophthalmol Vis Sci* **2017**, *58*, 2510-2519, doi:10.1167/iovs.16-20797.
7. Burdon, K.P.; Macgregor, S.; Hewitt, A.W.; Sharma, S.; Chidlow, G.; Mills, R.A.; Danoy, P.; Casson, R.; Viswanathan, A.C.; Liu, J.Z., et al. Genome-wide association study identifies susceptibility loci for open angle glaucoma at TMCO1 and CDKN2B-AS1. *Nat Genet* **2011**, *43*, 574-578, doi:10.1038/ng.824.
8. Abu-Amero, K.K.; Kondkar, A.A.; Mousa, A.; Almobarak, F.A.; Alawad, A.; Altuwaijri, S.; Sultan, T.; Azad, T.A.; Al-Obeidan, S.A. Analysis of Cyclin-Dependent Kinase Inhibitor-2B rs1063192 Polymorphism in Saudi Patients with Primary Open-Angle Glaucoma. *Genet Test Mol Biomarkers* **2016**, *20*, 637-641, doi:10.1089/gtmb.2016.0140.

9. Shiga, Y.; Nishiguchi, K.M.; Kawai, Y.; Kojima, K.; Sato, K.; Fujita, K.; Takahashi, M.; Omodaka, K.; Araie, M.; Kashiwagi, K., et al. Genetic analysis of Japanese primary open-angle glaucoma patients and clinical characterization of risk alleles near CDKN2B-AS1, SIX6 and GAS7. *PLoS One* **2017**, *12*, e0186678, doi:10.1371/journal.pone.0186678.
10. Burdon, K.P.; Crawford, A.; Casson, R.J.; Hewitt, A.W.; Landers, J.; Danoy, P.; Mackey, D.A.; Mitchell, P.; Healey, P.R.; Craig, J.E. Glaucoma risk alleles at CDKN2B-AS1 are associated with lower intraocular pressure, normal-tension glaucoma, and advanced glaucoma. *Ophthalmology* **2012**, *119*, 1539-1545, doi:10.1016/j.ophtha.2012.02.004.
11. Takamoto, M.; Kaburaki, T.; Mabuchi, A.; Araie, M.; Amano, S.; Aihara, M.; Tomidokoro, A.; Iwase, A.; Mabuchi, F.; Kashiwagi, K., et al. Common variants on chromosome 9p21 are associated with normal tension glaucoma. *PLoS One* **2012**, *7*, e40107, doi:10.1371/journal.pone.0040107.
